# Prevention of Aflatoxin B_1_-Induced DNA Breaks by β-D-Glucan

**DOI:** 10.3390/toxins7062145

**Published:** 2015-06-11

**Authors:** Eduardo Madrigal-Bujaidar, José Antonio Morales-González, Manuel Sánchez-Gutiérrez, Jeannett A. Izquierdo-Vega, Alicia Reyes-Arellano, Isela Álvarez-González, Ricardo Pérez-Pasten, Eduardo Madrigal-Santillán

**Affiliations:** 1 Conservation Medicine Laboratory, Superior School of Medicine, IPN. “Unidad Casco de Santo Tomas”. Plan de San Luis y Díaz Mirón. México, DF 11340, Mexico; E-Mail: jmorales101@yahoo.com.mx; 2 Genetics Laboratory, National School of Biological Sciences, IPN. “Unidad A. López Mateos”. Av. Wilfrido Massieu. Zacatenco, México, DF 07738, Mexico; E-Mails: eduardo.madrigal@lycos.com (E.M.-B.); isela.alvarez@gmail.com (I.A.-G.); 3 Institute of Health Sciences, Autonomous University of Hidalgo State, Ex-Hacienda de la Concepción, Tilcuautla, Hidalgo 42160, Mexico; E-Mails: spmtz68@yahoo.com.mx (M.S.-G.); jizquierdovega@gmail.com (J.A.I.-V.); 4 Organic Chemistry Department, National School of Biological Sciences, IPN. “Unidad Casco de Santo Tomas”. Carpio y Plan de Ayala. México, DF 11340, Mexico; E-Mail: areyesarellano@yahoo.com.mx; 5 Preclinical Toxicology Laboratory, National School of Biological Sciences, IPN. “Unidad A. López Mateos”. Av. Wilfrido Massieu. Zacatenco, México, DF 07738, Mexico; E-Mail: pastenrich@yahoo.com.mx

**Keywords:** aflatoxin B_1_, glucan, mouse hepatocytes, antigenotoxicity

## Abstract

Aflatoxins are a group of naturally-occurring carcinogens that are known to contaminate different human and animal foodstuffs. Aflatoxin B_1_ (AFB_1_) is the most genotoxic hepatocarcinogenic compound of all of the aflatoxins. In this report, we explore the capacity of β-d-glucan (Glu) to reduce the DNA damage induced by AFB_1_ in mouse hepatocytes. For this purpose, we applied the comet assay to groups of animals that were first administered Glu in three doses (100, 400 and 700 mg/kg bw, respectively) and, 20 min later, 1.0 mg/kg of AFB_1_. Liver cells were obtained at 4, 10 and 16 h after the chemical administration and examined. The results showed no protection of the damage induced by AFB_1_ with the low dose of the polysaccharide, but they did reveal antigenotoxic activity exerted by the two high doses. In addition, we induced a co-crystallization between both compounds, determined their fusion points and analyzed the molecules by UV spectroscopy. The data suggested the formation of a supramolecular complex between AFB_1_ and β-d-glucan.

## 1. Introduction

Aflatoxicosis is a condition caused by aflatoxins in both humans and animals [1]. Aflatoxins were first isolated some 40 years ago after outbreaks of disease and death in turkeys and of cancer in rainbow trout fed on rations formulated from peanut and cottonseed meals. These toxins belong to a group of compounds known as difurocoumarins, which are produced as secondary metabolites by the filamentous fungi *Aspergillus flavus* and *Aspergillus parasiticus*, which are widespread in nature [2,3]. These metabolites may produce considerable economic losses by attacking different stages of sowing and industrialization of different agricultural and dairy products. They can contaminate a great number of crops used for human and animal consumption, for example corn, peanut, sorghum, rice, wheat and nut, as well as various milk-made products [4]. In Mexico and other countries, corn is a cereal used as the main component of several meals. Cereals and other crops are exposed to fungal contamination in the field or during storage, resulting in mycotoxin contamination, and several studies have shown that these grains contain different levels of aflatoxin, particularly aflatoxin B_1_ (AFB_1_) [5,6]. Its mutagenic effects have been well documented in a number of *in vitro* and *in vivo* models, where the presence of DNA adducts, DNA breaks, gene mutations, induction of DNA synthesis and inhibition of DNA repair have been determined, as well as increases in the rate of chromosomal aberrations, micronuclei and sister chromatid exchanges (SCE) [3,7,8]. AFB_1_ is also a strong (Class I) carcinogen in mammalian species, where its exposure can give rise to different types of tumors, particularly in the liver [1]. A number of strategies have been tried to minimize the economic and biological problems raised by AFB_1_ contamination, including the application of adsorbents, heat, irradiation or chemical inactivation [3,9,10]. Furthermore, the experimental use of antigenotoxic agents has been assayed; with regard to this last aspect, the beneficial effects of probiotics have been evaluated. We have verified a protective activity of *Saccharomyces cerevisiae* in mice fed with AFB_1_-contaminated corn for six weeks and treated at the same time with the yeast; our results showed an inhibition as high as 70% of the micronuclei and SCE induced by the mycotoxin [11]. Such an effect was related to the presence of polysaccharides in the yeast cell wall. In fact, a number of investigations on this type of chemical, including β-d-glucan (Glu), α-mannan (Man) and glucomannan (Glucoman), has shown interesting beneficial properties related to their antigenotoxic, antioxidative, immunomodulating, anti-infective and antitumoral capacities [3,12]. β-d-glucan, in particular, is a highly branched oligosaccharide constituted by a main chain of glucoses with links α-1,6 and ramifications β-1,2 and β-1,3. Its chemical structure is very similar to α-mannan, which, in a previous study, administered during four weeks in mice fed with AFB_1_-contaminated corn, reduced the frequency of micronuclei and SCE by about 70% [13]. Other authors have demonstrated a significant *in vitro* antigenotoxic effect of the β-d-glucans obtained from the mushroom *Agaricus brasiliensis* in the reduction of DNA damage induced by B[*a*]P-7,8-dihydrodiol-9,10-epoxide (the main metabolite of B[*a*]P) and H_2_O_2_ in human peripheral lymphocytes [14].

The aim of the present study was to determine whether β-d-glucan can prevent the DNA damage produced by AFB_1_ in mouse hepatocytes. Another aim was to explore whether the antigenotoxicity of Glu could be related to the formation of a chemical complex with the mutagen.

## 2. Results

### 2.1. Antigenotoxic Effect of β-d-Glucan

**Figure 1:**
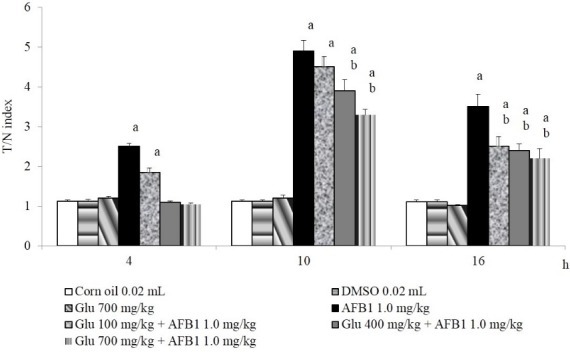
Antigenotoxic effect of β-d-glucan (Glu) against the DNA damage induced by aflatoxin B_1_ (AFB_1_) in mouse hepatocytes. Results are the mean ± SD of five mice per group (100 nuclei per doses). a, statistically-significant difference with respect to the value of the control groups and; b, with respect to the value obtained in mice treated with AFB_1_ only. ANOVA and Student–Newman–Keuls tests, *p* ≤ 0.05.

Figure 1 shows the comet measurements obtained in our assay. To summarize, at the fourth h of the schedule, no significant DNA damage induced by DMSO and β-d-glucan was found; therefore, these mice had a mean T/N index of 1.1. On the contrary, the animals treated with AFB_1_, as well as those administered with 100 mg/kg of Glu plus the mutagen had a statistically-significant DNA break increase. At 10 h, we determined a similar behavior regarding the control and the animals treated with β-D-glucan. In mice receiving only AFB_1_, a T/N index increase of about four times was calculated. With respect to the groups treated with the combination of chemicals, no protection was observed when the low dose of Glu was applied. However, a clear antigenotoxic effect was found with the two high doses; particularly with 700 mg/kg of Glu, the prevention of DNA damage was about 40%. Then, at 16 h, the genotoxicity of AFB_1_ and the protection exerted by Glu continue to be observed, but to a lesser extent.

### 2.2. Degrees of Damage Assessed by the Comet Assay

Table 1 shows the percentages describing the grades of damage determined in the experiment. The data agree with the comet measurements presented earlier. Nucleoids of control animals, as well as those treated with Glu alone corresponded mostly to Grade 0; however, nucleoids of mice treated with the mutagen and with the low dose of Glu plus AFB1 had a significant increase of Grades 2 and 3, but with the high dose of Glu plus AFB1, they showed more than a 40% reduction in the rate of such grades of damage.

**Table 1:** Grades of damage determined in the hepatocytes of mice treated with β-d-glucan (Glu) and with aflatoxin B_1_ (AFB_1_).

Agent/Dose	Time (h)	Grades of Damage (%)			
		G0	G1	G2	G3
Corn oil 0.02 mL	4	88	7	5	1
	10	91	7	1	1
	16	92	4	3	1
DMSO 0.02 mL	4	89	7	3	1
	10	86	11	2	1
	16	92	5	3	0
Glu 700 mg/kg	4	90	5	4	1
	10	91	5	4	0
	16	93	3	3	1
AFB1 1.0 mg/kg	4	69	11	10	10
	10	7	6	26	61
	16	18	16	28	38
Glu + AFB1 100 + 1.0 mg/kg	4	69	15	8	8
	10	11	16	24	49
	16	30	14	23	33
Glu + AFB1 400 + 1.0 mg/kg	4	76	9	6	9
	10	21	19	21	39
	16	33	18	23	26
Glu + AFB1 700 + 1.0 mg/kg	4	80	7	8	5
	10	19	22	27	32
	16	26	32	20	22

The grades of damage correspond to: G0, intact nuclei with no DNA displacement; G1, comets with a length no more than half of the nuclear diameter; G2, comets with no more than the length of one nuclear diameter; G3, comets with more than one nuclear diameter.

### 2.3. Melting Points of the Crystals Formed by the Compounds

Data corresponding to the melting point of the obtained crystals are presented in Table 2. Crystals formed by AFB_1_ plus Glu exhibited an intermediate value in comparison with the melting point of the independent compounds. The melting point of the joined compounds was higher than that of AFB_1_, but lower than that of Glu.

**Table 2:** Melting points of the crystals formed by aflatoxin B_1_ (AFB_1_) plus β-d-glucan (Glu) and of the independent compounds.

Chemical	Melting Point (°C)
AFB_1_/Glu	180.5
AFB_1_	132.5
Glu	240.0

**Figure 2:**
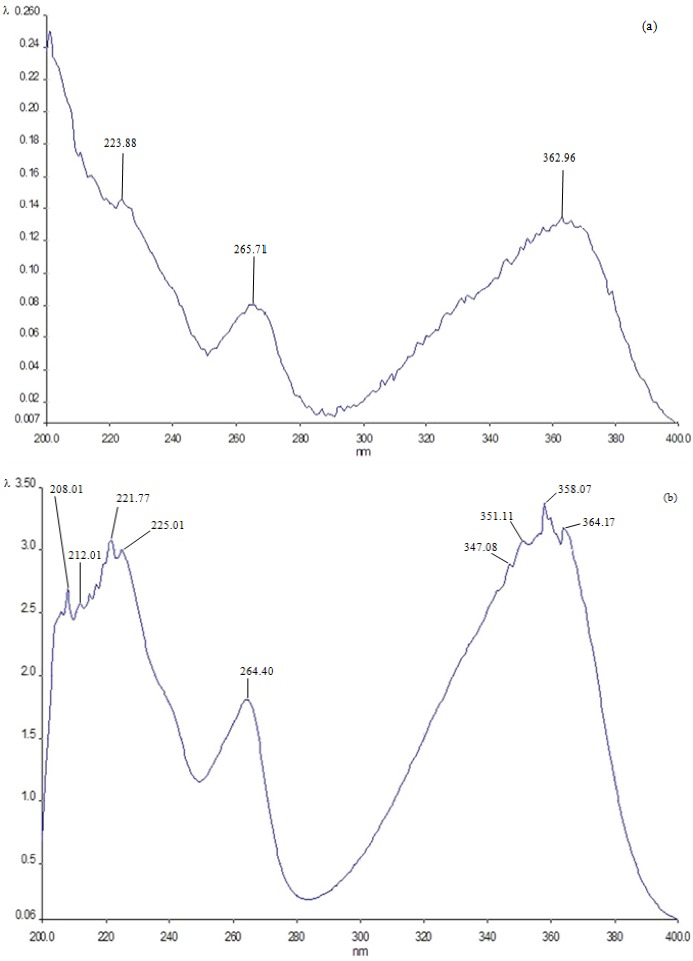
UV spectrum of the crystals formed by β-d-glucan (Glu) plus AFB_1_ (**a**) and that corresponding to AFB_1_ (**b**). The λ values in the ordinate of (**a**) vary from 0.007 to 0.24; in (**b**), the values are from 0.06 to 3.50. The maximum peaks in (**a**) (224, 266 and 363 nm) show a correspondence with those detected in (**b**) (222, 264 and 358 nm).

### 2.4. UV Spectrum Analysis of the Crystals Formed by the Compounds

**Figure 3:**
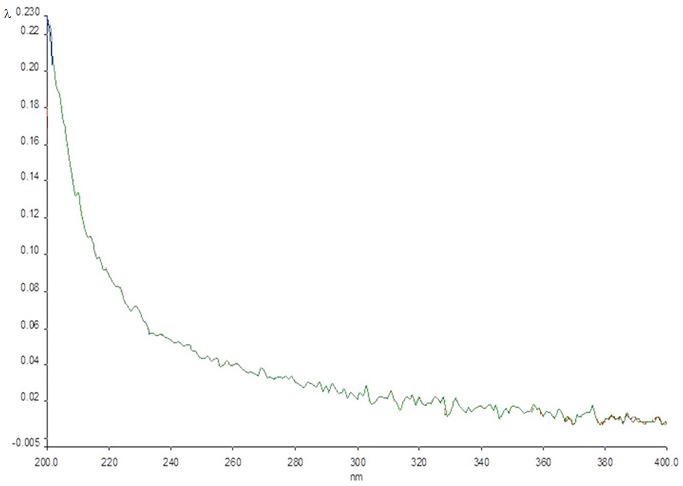
UV spectrum of the crystals formed by β-d-glucan (Glu). The λ values in the ordinate vary from 0.005 to 0.230. No peaks were found in the spectrum.

Figure 2a shows the UV spectrum of the crystals obtained. The presence of AFB_1_ in the mixture is clear, as indicated by its characteristic maximum peaks at 224, 266 and 363 nm, which were very similar to those detected in the spectrum of the mutagen in the independent form (Figure 2b). Moreover, the spectrum obtained with the crystals differs sharply from that obtained with the polysaccharide alone, which showed no peaks in the range from 220 to 400 nm (Figure 3).

## 3. Discussion

The present study demonstrates the utility of the single-cell gel electrophoresis assay to evaluate the genotoxic and antigenotoxic capacities of the compounds used. As in other studies, we confirmed that it is a simple, sensitive and rapid technique for damage detection within genetic material at an early stage, coinciding with other authors, considering that this technique also detects DNA single- and double-strand breaks, alkaline labile sites (ALS), oxidative DNA damage, DNA cross-links, DNA adducts, apoptosis and necrosis [15,16,17,18,19,20]. With respect to the dose of AFB_1_ (1.0 mg/kg) used in this study, there are also several studies where different doses ranging from 250 μg/kg up to 9 mg/kg were used. This wide range of doses is attributed to the fact that mice are considered as a species resistant to the toxic effects of AFB_1_, since they have a high activity in glutathione-*S*-transferase [21]. However, evidence also suggests that its toxic effect in rodents depends on the strain, route of administration, time of exposure and the organ or tissue where such toxicity is evaluated. Our results demonstrated that the dosage used was sufficient to produce DNA damage on hepatocytes and also that the maximum break was at Hour 10 with respect to all other periods tested, which is consistent with data obtained from an acute comet assay with a single administration [22], which establishes that the liver is one of the main organs affected after oral administration. Besides, due to the assay detection of the repair process [22], in our case, the results suggest that it may begin after 10 h of exposure to chemicals. In general, the experimental model and the dose used coincide with those reported in other studies and support the observations made in previous research conducted by our research group, where the α-mannan capacity is explored to reduce the DNA damage induced by AFB_1_ [20,21,22,23,24,25,26,27,28].

Furthermore, there is experimental and epidemiological evidence that compounds of natural origin, including polysaccharides, have the ability to protect against mutagenic damage. Moreover, it is also known that the cell wall of yeast is composed of complex polymers β-d-glucans, α-mannans, α-mannoprotein and a minor amount of chitin, all of which have a number of bioprotective properties [29]. In the specific case of glucan, this polysaccharide has caught scientific attention, mainly due to its immunomodulatory capacity, which has been demonstrated in some studies to decrease the immunosuppressive actions of various immunotoxins, including mercury and perluoroctanic acid [30]. Similarly, field studies related to his genotoxicity, antigenotoxicity and chemoprevention capacity have increased. Its ability to partially prevent DNA damage induced by AFB_1_ in mouse hepatocytes was also determined in this assay, which confirms what other scientists have mentioned, that β-d-glucan lacks genotoxic or systemic toxicity [31]; on the contrary, the results suggest that it is a genoprotector agent, whose potential is dose-dependent, for the best response was favored at higher doses. In this context, other authors have used the comet assay in the human hepatoma cell line (HepG2) in order to determine the chemopreventive effect of β-d-glucan against benzo[a]pyrene [32] and found similar results to those reported in this study *in vivo*.

Regarding polysaccharides, including β-d-glucan and α-mannan, it has been proposed that their antigenotoxic action mechanism is related to their action as antioxidant agents. This activity is suggested in various methods *in vitro* and *in vivo*, where the mutagenicity induced by acetaminophen, cyclophosphamide, adriamycin, cisplatin and ofloxacin has been evaluated [33,34,35,36,37,38]. However, our results suggest another chemopreventive mechanism, which is related to its adsorbing effect. With regard to this mechanism, a number of epidemiological reports have suggested that a low-fat, high-fiber diet is beneficial for the prevention of cardiovascular disease, as well as of colon and breast cancer, in the latter cases probably by absorbing carcinogens and promoters and increasing stool bulk to facilitate their elimination [39,40].

Preclinical studies have also revealed that phytochemicals act to delay, block or reverse carcinogenesis, as in the case of coffee fiber (arabino-galactose polymer) and pectin, which significantly reduced the rate of azoxymethane-induced aberrant colonic crypts in rat [41]. Likewise, wheat bran arabinoxylans have shown the induction of the detoxifying enzyme glutathione *S*-transferase and reduction of genotoxicity by hydrogen peroxide and 4-hydroxynonenal in HT29 colon cancer cells [42]. AFB_1_ mutagenicity is related to the metabolic activation by CYP3A4, CYP3A5 and/or CYP1A2 to form the exo-8,9 epoxide, which is highly reactive and binds to the *N*-7 position of guanine residues in DNA [16]. Specifically in developing countries, AFB_1_ contamination can be a major health problem due to discontinuous monitoring and inefficient storage conditions, as evidenced by the development of tumors that seriously affect different countries [4]. This is compelling evidence that decreasing such contamination is important. In the experimental conditions of the present study, the comet assay detected genotoxic damage produced by the mutagen after its rapid absorption by the small intestine and its transportation to the liver cells by the mesenteric blood, as well as residual DNA damage that β-d-glucan (which is not absorbed in the intestine) was unable to eliminate from the digestive tract, probably by an incomplete binding between the mycotoxin and oligosaccharide. Some *in vitro* studies have shown that β-d-glucan could bind ABF_1_, suggesting a reduction of bioavailability in the digestive tract [43,44]; however, the absorption capacity may be limited in micromolar concentration ranges of AFB_1_. Due to the difficulty in assessing the mechanism by which Glu could bind aflatoxin in the intestine of mice, we have now investigated in an *in vitro* model in which the β-d-glucan forms a chemical complex with the mycotoxin. In this respect, β-d-glucan probably wraps the mutagen, and both compounds constitute a supramolecular complex. We suggest this protective mechanism of β-d-glucan, because, considering the melting point, which is a criterion of chemical purity, we detected an intermediate value in the AFB_1_-β-d-glucan crystals in comparison to the value obtained for the independent compounds; other reasons are that the spectrum of the AFB_1_-β-d-glucan crystals does not correspond to the characteristics of β-d-glucan and that the melting point data show that the crystal was not formed solely by AFB_1_. Interestingly, the spectrum AFB_1_-β-d-glucan apparently did not show changes in the mycotoxin structure. These data are consistent with the formation of a supramolecule, where the subunits are linked by noncovalent bonds and where the chemical architecture may be supported by weak interactions, such as hydrogen, C–H ... π, π–π bonds, among others [45,46,47]. In our study, the –OH groups from the polysaccharide (which is an H donor) may perhaps be joined to the O atoms of AFB1, especially to the vicinal C=O groups, thereby forming a cyclic supramolecular synthon.

Finally, the crystallization between the mycotoxin and β-d-glucan shows the formation of the supramolecular complex with adsorptive capacity; in this sense, there is evidence that glucomannan (polysaccharide similar to Glu) has the ability to adsorb the AFB_1_ and T-2 toxin in the gastrointestinal tract of broiler chickens [48]. Furthermore, it reduces liver cholesterol by a viscosity-mediated interference of cholesterol absorption [49]. This adsorbent capacity may be considered an extracellular mechanism, similar to dietary fiber, for inhibiting the penetration of carcinogens and removing them from the organism [50].

## 4. Experimental Section

### 4.1. Chemicals and Animals

The following compounds were purchased from Sigma Chemicals (St. Louis, MO, USA): AFB_1_, dimethyl sulfoxide (DMSO), Triton X-100, disodium sulfate, disodium ethylenediamine (EDTA), low melting point agarose (LMPA), ethidium bromide, Trizma base, phosphate buffer saline (PBS), sodium chloride, sodium hydroxide, methanol, ethanol (HPLC grade), calcium chloride and disodium sulfate. Trypan blue and normal melting point agarose (NMPA) were obtained from Invitrogen-Gibco (Carlsbad, CA, USA). β-d-glucan was donated by the Institute of Chemistry, Bratislava (Slovakia), and was prepared from the cell wall of *S. cerevisiae* [51]. It is a branched β-(1,3)-glucan containing β-(1,6)-glucosidic inter-chain linkages and has a purity of 96%.

The experiment was done using eleven-week-old NIH male mice weighing 20 ± 2.0 g, which were obtained from CENID-Microbiology, a department of the Mexican Ministry of Agriculture. The animals were maintained at 23 °C in polypropylene cages (five individuals per cage) with heat-treated hard wooden bedding, in a 12-h dark-light cycle and 50% ± 10% humidity. They were allowed to freely consume tap water and Purina rodent food. The experimental protocol was approved by the Committee of Ethics and Biosecurity at the National School of Biological Sciences.

### 4.2. Genotoxicity/Antigenotoxicity Protocol

#### Experimental Design

AFB_1_ was dissolved in DMSO/corn oil (1:1) and β-d-glucan (Glu) in distilled water. All compounds were administered in a few seconds, without anesthesia, using an intragastric cannula to the following seven groups constituted by 15 individuals each: (1) a group of mice administered with 0.02 mL of corn oil; (2) a group treated with 0.02 mL of DMSO; (3) a group treated with 700 mg/kg bw of Glu; (4) one more group treated with 1.0 mg/kg of AFB_1_; and (5) three groups that were first administered Glu (100, 400 and 700 mg/kg, respectively) and, 20 min later, 1.0 mg/kg of AFB_1_.

The administered doses were selected on the basis of preliminary assays that evaluated the potential genotoxicity and systemic toxicity of the involved chemicals. Results obtained in animals treated with DMSO/corn oil (1:1) were similar to those determined with the independent compounds.

At 4, 10 and 16 h post-administration, five mice per group were cervically dislocated and dissected to obtain their liver in iced PBS; a fraction of the organ was then macerated to obtain a cell suspension, which was adjusted to about 10,000 cells/mL. The trypan blue method was used to determine the number of viable cells, which was always more than 85%.

### 4.3. Unicellular Alkaline Electrophoresis (Comet) Assay

We followed a previously reported method [24,52]. First, we deposited normal melting point agarose (110 μL) on a fully-frosted slide, and onto this layer, we then put a mixture of the cell suspension (10 μL) plus LMPA (75 μL); finally, we added another layer of low melting point agarose (75 μL). After a few minutes, the mixture solidified, and the slide was placed at 4 °C in a Coplin jar containing the lysis solution (NaCl 2.5 M, EDTA 100 mM, Trizma base 10 mM, 1% Triton^®^ X-100 and 10% DMSO). Twenty-four hours later, the nuclei were placed in alkaline buffer (NaOH 30 mM and EDTA 1 mM, pH > 13) for 30 min and then in an electrophoresis chamber for 20 min at 300 mA, 23 V and pH > 13. Slides were subsequently washed three times (5 min each) with a 0.4 M Trizma solution made in deionized water, pH 7.5, to neutralize the earlier process, and dried at room temperature. Finally, the nucleoids were stained with ethidium bromide (50 μL).

Observations were made at 400× in an epifluorescent microscope (Axiophot-1, Carl Zeiss, Texas A&M University, College Station, TX, USA) equipped with a digital camera (ZWS-47DE) and a program for capturing, processing and analyzing images (Carl Zeiss, KS400 Version 3.01). In 100 nucleoids per dose/time, we recorded the extent of DNA damage caused by the mutagen and the protection exerted by the polysaccharide. For this purpose, we obtained the length-to-width index (T/N index) measuring the image length and dividing the result by the head diameter [24,53]. The data were statistically analyzed with ANOVA followed by the Student–Newman–Keuls test, using the GraphPad Instat program, Version 2 (for Windows). In addition, we determined the percentage of cells without DNA migration and those with migration according to 4 grades of damage, where Grade 0 corresponded to intact nuclei with no DNA displacement, Grade 1 to comets with a length no more than half of the nuclear diameter, Grade 2 to no more than the length of one nuclear diameter and Grade 3 to more than one nuclear diameter.

### 4.4. Crystallization, Melting Point Determination and Spectroscopic Analysis

The tested chemicals were co-crystallized following preliminary assays to determine the appropriate experimental conditions. Based on these results, we dissolved AFB_1_ (5 mg) in HPLC-grade ethanol (5 mL) and Glu in a mix of ethanol-deionized water (1:1). Then, each solution (1 mL) was mixed in the dark (at 26 °C, pH = 7.0); crystals were formed for 11 days by means of the hanging drop technique [54]. The obtained uncolored crystals were dried with CaCl2 for 72 h; after that, their melting point was obtained twice using electrothermal equipment (Barnstead/Thermoline). Finally, the remaining crystals were dissolved in ethanol-deionized water (1:1) and UV analyzed in the range 200 to 400 nm with a Perkin Elmer Lambda 19 spectrophotometer.

## 5. Conclusions

In the present investigation, we demonstrated the capacity of Glu to protect from the DNA damage induced by AFB_1_ in mouse hepatocytes and, consequently, the potential preventive effect of the compound against cancer development. Moreover, we provided evidence that suggests that such an effect was due to a supramolecular complex formed between the two involved compounds. This information confirms previous reports on the bioprotective effects of Glu, as well as studies on other polysaccharides of the cell wall of yeasts. However, our data also suggest the need to explore other experimental conditions, so as to improve the observed effect of Glu and to determine this capacity on damaged colon cells. This is so, moreover, because the interaction of the tested compounds with other molecules of the diet can influence the effect in this organ. Besides, it seems pertinent to extend *in vivo* studies on the matter in regard to all yeast polysaccharides, as well as to perform long time assays.
